# Survey results of 3D‐CRT and IMRT quality assurance practice

**DOI:** 10.1002/acm2.12885

**Published:** 2020-04-30

**Authors:** Hunter Mehrens, Paige Taylor, David S. Followill, Stephen F. Kry

**Affiliations:** ^1^ Imaging and Radiation Core Houston QA Center Houston TX USA; ^2^ Department of Radiation Physics The University of Texas M. D. Anderson Cancer Center Houston TX USA

**Keywords:** dose verification, IMRT QA, patient specific QA, survey, TG‐218

## Abstract

**Purpose:**

To create a snapshot of common practices for 3D‐CRT and intensity‐modulated radiation therapy (IMRT) QA through a large‐scale survey and compare to TG‐218 recommendations.

**Methods:**

A survey of 3D‐CRT and IMRT QA was constructed at and distributed by the IROC‐Houston QA center to all institutions monitored by IROC (n = 2,861). The first part of the survey asked about methods to check dose delivery for 3D‐CRT. The bulk of the survey focused on IMRT QA, inquiring about treatment modalities, standard tools used to verify planned dose, how assessment of agreement is calculated and the comparison criteria used, and the strategies taken if QA fails.

**Results:**

The most common tools for dose verification were a 2D diode array (52.8%), point(s) measurement (39.0%), EPID (27.4%), and 2D ion chamber array (23.9%). When IMRT QA failed, the highest average rank strategy utilized was to remeasure with the same setup, which had an average position ranking of 1.1 with 90.4% of facilities employing this strategy. The second highest average ranked strategy was to move to a new calculation point and remeasure (54.9%); this had an average ranking of 2.1.

**Conclusion:**

The survey provided a snapshot of the current state of dose verification for IMRT radiotherapy. The results showed variability in approaches and that work is still needed to unify and tighten criteria in the medical physics community, especially in reference to TG‐218's recommendations.

## 1 INTRODUCTION

With the complex nature and new adaptations of intensity‐modulated radiation therapy (IMRT), there is a high need for robust quality assurance (QA). This is particularly true as Imaging and Radiation Oncology Core (IROC) phantoms continue to show serious discrepancies between delivered and planned doses in IMRT treatments at a large number of institutions.^1^, ^2^ Molineu et al. showed a pass rate of only 81.6% for head and neck phantoms between 2001 and 2011.^1^ These errors are most often manifesting as systematic dose errors (>58%) with the highest failure rate due to underdosing,^2^ and deficiencies in beam modeling has been implicated as a widespread cause of this problem with 17% of passing phantoms and 68% of failing phantoms being affected by calculation errors.^3^

While novel approaches to improve QA, such as deep learning, are being developed and tested,^4^, ^5^, ^6^ routine measurement based approaches remain the standard. Recent comparisons of VMAT to routine IMRT QA have also shown that as new techniques are developed, there is also a need for new QA techniques.^7^, ^8^, ^9^, ^10^, ^11^, ^12^, ^13^, ^14^, ^15^, ^16^ These papers show the importance of understanding the current QA procedures of the medical physics community to ensure that progress is being made toward more robust and uniform management of IMRT QA.

Nelms and Simon surveyed several hundred sites about their planar IMRT QA methods and analysis, which helped identify how IMRT QA was evolving.^17^ However, the scope of their survey was narrow and is now 12 years old.^17^ Similarly, a survey of eight vendors was included in the TG‐218 study to gain information on what was the current state of IMRT QA practice; but again, the scope was limited to specific aspects of IMRT QA, for example, gamma analysis and calculation (absolute vs relative), following the focus of the Nelms and Simon study.^18^ With the recently published TG‐218 guidelines for clinical IMRT QA, a large encompassing survey could shed light on how current IMRT QA practices compare to the TG‐218 recommendations.

To this end, a survey was created to broadly assess the current practice of IMRT QA, and at the same time capture other QA information, including MU verification for 3D‐CRT. With this survey including multiple facets of QA, a baseline of common practices can be made known to the community. Our survey examined these facets of current QA practices and thereby provides a snapshot of the QA world for the medical physics community.

## 2 METHODS

The IROC Houston QA Center monitors sites that participate in NCI‐sponsored trials through several audit processes that include annual output checks and anthropomorphic phantom irradiations. To track changes in each site's personnel, machines, and treatment modalities, IROC maintains an electronic Facility Questionnaire. This questionnaire is sent annually (or more often as needed) to every institution to allow for updates to the institution's status.

A QA survey was created and included in IROC‐Houstons Facility Questionnaire that was open from August 2011 to January 2018. Our data includes only those sites that had updated their Facility Questionnaire in 2017 in order to capture the most up‐to‐date information. The survey had two main sections: 3D‐CRT MU verification and IMRT QA. For all sections, standard/common answers were provided, while open‐ended “other” options were also available for less common/unexpected answers.

Table 1, shows the questions and possible responses pertaining to 3D‐CRT verification of MU. This section was almost exclusively open‐ended in response. Table 2 shows the main questions and the varied possible responses for the largest section of the survey on IMRT QA.

**Table 1:** Verification of delivered dose for 3D‐CRT.

Questions	Available Answers
Describe the method(s) used to conduct a check of the dose and monitor unit calculations generated by the 3DRTP system:	Open Ended
Are your 3D‐CRT treatments monitored by a record and verify system?	Yes (Manufacturer & Model) No

**Table 2:** Patient‐specific QA (IMRT QA): verification of delivered dose.

Questions	Available answers
Which of the following treatment modalities does you institution use? (Check all that apply)	Routine IMRT (Sliding Window, Step and Shoot, Tomotherapy etc.) VMAT/Rapid Arc
What are your standard tool(s) for verifying that the treatment unit delivers the planned dose for individual patients? (Choose all that apply.)	Point(s) Measurement Film 2D Diode array 2D Ion Chamber array EPID 2.D (pseudo 3D) array/multi‐plane array 3D dosimeter Other
When you make QA measurements, which of the following do you most commonly do?	Deliver beams at the same fixed gantry angle Deliver at the planned gantry angle
Do you mount your detector on the gantry?	Yes No
Are your plans usually assessed for pass or fail based on:	Each field‐by‐field measurement Composite measurement (all fields)
How do you assess agreement (select all that apply), and what are your most commonly used comparison criteria?	Point Dose Planar 3D/Volumetric analysis
Do you do routine in‐vivo dosimetry for IMRT patients?	Yes No
If your QA does not meet your passing criteria, what actions do you take? (choose all that apply, rank in order of attempt (1 denotes first strategy))	Remeasure with the same setup (at the same point/plane) Move to a new calculation point/plane and remeasure Try fixed gantry angle delivery Re‐plan Scale the MU's (partially or fully) Change the passing criteria for the case Analyze in relative dose mode instead of absolute dose mode Document result and deliver the plan Something else: __________

For many of the primary questions shown in Table 2, there were secondary follow‐up questions related to the original question. For example, the question “How do you assess agreement (select all that apply), and what are your most commonly used comparison criteria?” had three answers associated with it: Point Dose, Planar, and 3D/Volumetric analysis. Each of these answers contained further questions about the specifics of each. For example, a response of “planar” assessment prompted secondary questions about the use of absolute vs relative dose, the number of planes used in assessment, and the test performed, including gamma criteria. The complete survey, primary and secondary question, with their possible responses, is listed in Data S1.

## 3 RESULTS

Through IROC‐Houston's extensive list of monitored institutions, the survey was made available to 2,681 sites. To be the most current, we excluded any site that had not updated its facility questionnaire in 2017 (the last year of the survey). The results from a total of 1,455 survey participants were analyzed. The majority (91.9%) of responding sites were from the United States and Canada.

### 3.A Verification of delivered dose for 3D‐CRT

Responses to the survey questions listed in Table 1: “Describe the method(s) used to conduct a check of the dose and monitor unit calculations generated by the 3DRTP system:” varied due to its open‐ended nature. However, majority of the responses indicated the use of a version of RadCalc (50.8%) for this procedure. Other responses included in‐house software, manual hand calculations, or other third party software.

In response to the question: “Are your 3D‐CRT treatments monitored by a record and verify system?”, an overwhelming majority (98.8%), but not all sites, confirmed their use of a record and verify system, with Aria (Varian Medical Systems; 50.4%) and Mosaiq (Elekta; 39.4%) being among the most prevalent choices.

### 3.B Patient‐specific QA (IMRT QA): verification of delivered dose

**Fig. 1:**
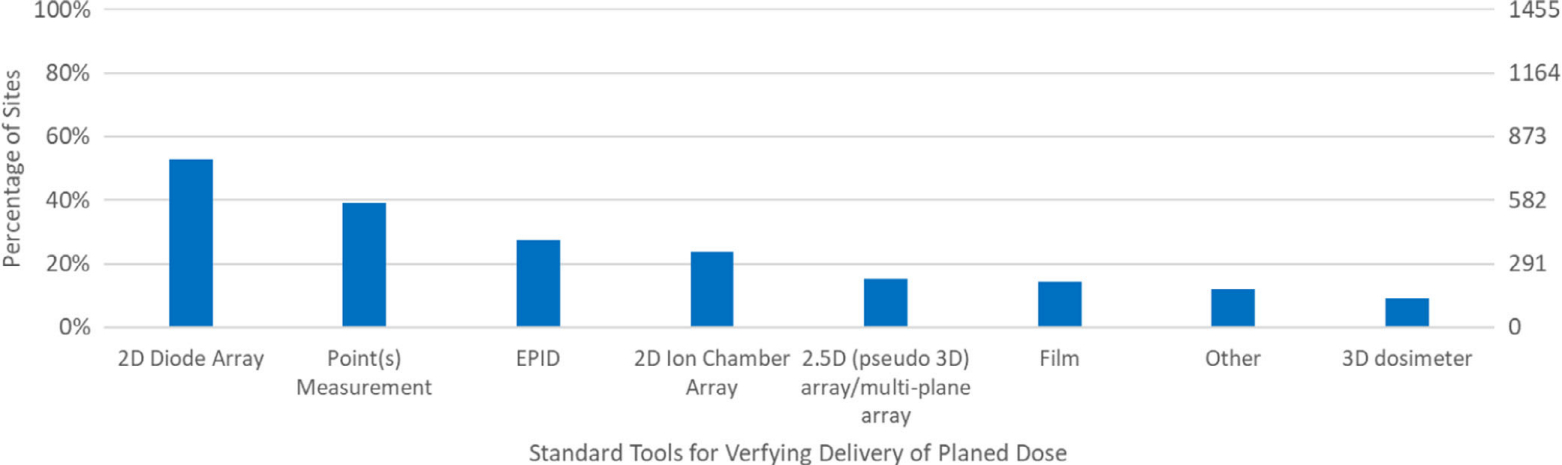
Responses to Survey Questions: “What are your Standard Tool(s) for Verifying that the Treatment Unit Delivers the Planned Dose for Individual Patients?”.

**Fig. 2:**
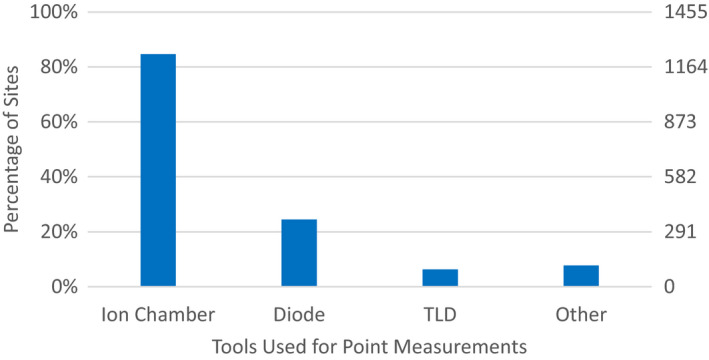
Responses to Survey Questions: “What are your Standard Tool(s) for Verifying that the Treatment Unit Delivers the Planned Dose for Individual Patients?” and choosing Point(s) Measurement for their standard tool.

**Fig. 3:**
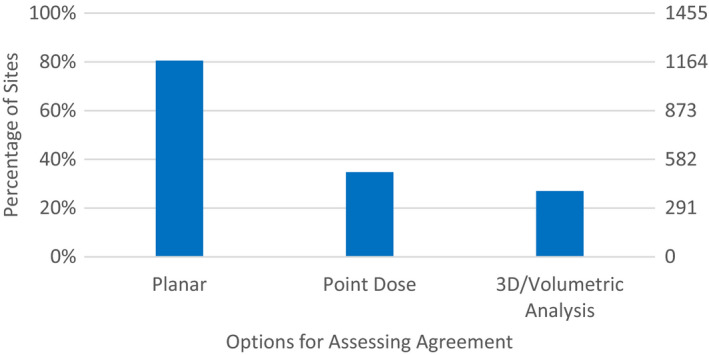
Response to Survey Questions: “How do you assess agreement, and what are your most commonly used comparison criteria?”.

Responses to the IMRT QA questions listed in Table 2 are shown in Figs. 1, 2, 3. A choice of Routine IMRT and/or VMAT/Rapid Arc was available with a follow‐up question inquiring if QA varied between the two choices, if applicable. A large portion of the sites responded that they conducted QA for both IMRT and VMAT treatments (69.3%), with 65.2% of these respondents not differing in QA practices between the two delivery modes.

Figure 1 shows that the most common tools for dose verification are 2D diode arrays (52.8%), point(s) measurements (39.0%), EPIDs (27.4%), and 2D ion chamber arrays (23.9%). Many sites used multiple devices; the number of standard tools utilized by sites was most often one (40.1%), but was commonly two (33.5%) and even three (18.5%). Respondents reported using up to seven different tools for IMRT QA.

Respondents who indicated that they used point measurements indicated that they primarily used an ion chamber (84.7%), with approximately equal distribution of several chamber volumes: radius less than 0.1 mm (33.1%), 0.1–0.2 mm (29.0%), or 0.6 −0.7 mm (27.7%). Diodes (24.5%) and TLD (6.3%) were also used, but less often. 7.7% of respondents indicated an “other” device was used, which was most commonly a MOSFET.

For array detectors, respondents were asked if they evaluated their measurement in the phantom geometry or if they mapped it onto the patient dataset (ie DVH analysis). The vast majority of the responses indicated that they evaluated their measurement in the phantom geometry as shown in Table 3.

**Table 3:** Response to survey question: “What comparison do you perform?” for a subset of standard tools (2D Diode Array, Ion Chamber Array, EPID, and 2.5D (pseudo 3D) Array/Multi‐plane Array) used for the verification of planned dose delivered by the treatment unit. N is the number of respondents who selected the tool and should vary with each tool as indicated.

	2D Diode Array (N = 853)	Ion Chamber Array (N = 426)	EPID (N = 421)	2.5D (pseudo 3D) array/multiplane array (N = 245)
Measurement vs Calculation in Phantom	97.9% (835)	96.5% (411)	87.4% (368)	92.2% (226)
Measurement Mapped onto Patient CT Dataset	2.1% (18)	3.5% (15)	12.6% (53)	7.8% (19)

When making QA measurements, 62.6% of sites deliver beams at the planned gantry angle, while 37.0% delivered beams at the same fixed angle. Of these fixed angle deliveries, the plans were either assessed through composite measurements (60.2%) or field‐by‐field measurements (39.3%). The overwhelming majority (86.1%) of sites do not mount their detector on the gantry.

The previous portions of the survey sought information about the specific devices used. The subsequent survey questions explored ways in which IMRT QA was analyzed. The next survey question inquired about how the IMRT QA tools (from Fig. 1) assessed agreement. As seen in Fig. 3, planar analysis (80.6%) was by far the most common followed by using point dose (34.7%) then 3D/volumetric analysis (27.0%).

The vast majority of sites using planar measurements to assess agreement utilize absolute dose (87.6%) as compared to relative dose (16.1%), with almost every site using gamma analysis (99.4%) as the evaluation metric. Table 4 provides the number of participants that used different passing criteria for their IMRT QA gamma analysis. The loosest criteria was 5%/5 mm with ≥ 90% pixels passing and the tightest criteria was 1%/2 mm with ≥ 90% pixels passing, indicating a large variability in passing criteria. The most common criteria used was 3%/3 mm (84.3%). For percentage of pixels passing, 95% (56.3%) and 90% (34.4%) shared the majority of responses from sites.

**Table 4:** Responses to survey questions: planar secondary questions for passing criteria for gamma analysis.

		Gamma distance‐to‐agreement			
		2 mm	3 mm	4 mm	5 mm
Gamma dose percent difference	1%	0.1% (1)*	0.1% (1)	0.0% (0)	0.0% (0)
	2%	5.1% (58)*	0.1% (1)	0.0% (0)	0.0% (0)
	3%	4.0% (46)*	84.3% (963)	0.1% (1)	0.1% (1)
	4%	0.1% (1)	0.1% (1)	0.7% (8)	0.0% (0)
	5%	0.2% (2)	1.0% (12)	0.0% (0)	1.9% (22)

The gamma distance‐to‐agreement ranged from 2 to 5 mm while the gamma dose percent difference ranged from 1 to 5%. Percentage based on number of sites that provided answers for the criteria (N = 1143). *Acceptable criteria based on recommendations for TG‐218

For those utilizing point dose, the majority of the sites only utilized one point for analysis (65.6%), although many places used more (eg 20.5% of respondents used three points). Acceptability criteria was most often 3% (47.3%) or 5% (39.3%), but ranged all the way from 1–10%.

Very similarly to planar evaluations, the vast majority of sites using 3D/Volumetric measurements to assess agreement utilize absolute dose (90.5%) as compared to relative dose (9.5%), with almost every site using gamma analysis (95.1%) as the test. Table 5 provides the number of participants that used a certain passing criteria for gamma analysis. The loosest criteria was 5%/5 mm with ≥ 90% pixels passing and the tightest criteria was 1%/3 mm with ≥ 97% pixels passing indicating, again, a large variability in passing criteria. Again, the most common criteria used was 3%/3 mm (84.1%). For percentage of pixels passing, 95% (54.2%) and 90% (37.1%) also shared the majority of responses from sites.

**Table 5:** Response to survey questions: 3d/volumetric analysis secondary questions for passing criteria for gamma analysis.

		Gamma distance‐to‐agreement			
		2 mm	3 mm	4 mm	5 mm
Gamma dose percent difference	1%	0.0% (0)*	0.3% (1)	0.0% (0)	0.0% (0)
	2%	5.4% (21)*	0.5% (2)	0.0% (0)	0.0% (0)
	3%	3.6% (14)*	84.1% (329)	0.0% (0)	0.5% (2)
	4%	0.0% (0)	0.0% (0)	0.8% (3)	0.0% (0)
	5%	0.0% (0)	1.5% (6)	0.0% (0)	0.5% (2)

The gamma distance‐to‐agreement ranged from 2 to 5 mm while the gamma dose percent difference ranged from 1 to 5%. Percentage based on number of sites that provided answers for the criteria (N = 391). *Acceptable criteria based on recommendations for TG‐218.

If IMRT QA did not pass, we provided nine possible follow‐up steps to choose from in our survey. Sites were given the opportunity to rank them on a scale of one to nine, with one denoting the first strategy used. These strategies are ordered in Table 6 according to the order of their average rank (for places employing that strategy). The highest average rank selection was to remeasure with the same setup, which had an average position ranking of 1.1 with 81.4% of sites placing this at rank one; 90.4% of facilities employ this strategy. The second highest average rank selection was to move to a new calculation point and remeasure (54.9%); this had an average ranking of 2.1. Strategies became less clearly established in the community after this: the third highest average rank selection was “other”, ie not one of the nine options provided.

**Table 6:** Response to survey questions: if your QA does not meet your passing criteria, what actions do you take? (Choose all that apply, rank in order of attempt (1 denotes first strategy)).

Strategies	Average Rank	Percentage of Sites (Number):	Rank 1	Rank 2	Rank 3	Rank 4	Rank 5	Rank 6	Rank 7	Rank 8	Rank 9
Remeasure with the Same Setup	1.1	90.4% (1316)	81.4%	7.0%	1.8%	0.2%	0.1%	–	–	–	–
Move to a New Calculation Point and Remeasure	2.1	54.9% (799)	4.1%	41.3%	7.6%	1.2%	0.3%	0.2%	–	–	–
Other	2.6	26.9% (391)	4.7%	9.1%	7.1%	4.3%	1.1%	0.5%	–	–	0.1%
Analyze in Relative Dose Instead of Absolute Dose	2.8	25.9% (376)	2.5%	8.1%	8.4%	4.9%	1.3%	0.4%	0.1%	0.1%	–
Try Fixed Gantry Angle Delivery	2.9	11.6% (169)	0.5%	3.3%	5.1%	1.7%	0.6%	0.1%	0.1%	–	–
Change the Passing Criteria for the Case	3.1	30.2% (440)	2.7%	7.5%	9.7%	6.3%	2.7%	0.7%	0.3%	0.2%	–
Replan	3.3	84.0% (1222)	2.3%	17.3%	34.0%	20.2%	8.3%	1.5%	0.5%	–	–
Scale the MU's	3.5	11.4% (166)	0.3%	2.3%	3.6%	2.8%	1.2%	0.7%	0.2%	0.2%	–
Document Result and Deliver Plan	4.3	17.4% (253)	0.3%	1.2%	3.1%	5.2%	3.9%	2.3%	0.7%	0.5%	0.1%

Percentages are based on 1455 participants.

Looking into the data further, we analyzed the second and third choices selected given a particular first choice. When the most common strategy, remeasure with the same setup, was selected with rank one, the top two most common selections for rank two were to move to a new calculation point and remeasure, followed by replan. In addition, replan was also the most common selection for rank three. This may indicate that these three strategies tend to be the most commonly used in the medical physics community. Additional strategies and their prevalence are shown in Table 6. Overall, a large majority selected at least one (98.8%) or two (97.0%) strategies, with a substantial fall off occurring at four (46.8%) to five (19.6%) strategies. Seventeen sites did not select any strategies.

## 4 DISCUSSION

Our survey captured many facets of MU verification for 3D‐CRT, and IMRT QA. For IMRT QA, our survey evaluated the use of different tools and methods to assess agreement between planned and delivered dose, and explored the strategies used to deal with failing plans. The results of our survey showed that a wide variety of tools and techniques are used for assessing agreement between measured and planned doses. One important note is that while we sometimes specify manufacturers that are used, we do not state any usefulness or correctness in their utilization. We only report these results as a snapshot of the community practice.

As with every survey, any biases must be understood before formulating conclusions. With the structure of the survey, sites were able to answer secondary questions without answering the primary question. For example, a site could select absolute dose or relative dose for a planar technique for agreement assessment, without actually selecting the planar option to begin with. This sometimes caused a discrepancy between our totals; however, these discrepancies were minimal. Another potential bias is the period over which the survey was available. IROC‐Houston's Facility Questionnaire contains more information than just this survey, and the specifics of what was updated in the Facility Questionnaire was not tracked (just the binary fact that it was updated). We have assumed that when a facility updated their Facility Questionnaire, they did this comprehensively, as is requested of the institutions. However, it is possible that this was not always done, which could lead to some institutional results being descriptive of a previous year. Finally, while the number of survey participants was large, our selectivity of participants in the survey only included sites that were actively participating in NCI‐sponsored trials, which focuses primarily on, and is therefore reflective of practice in, the United States and Canada. In addition, it is of note that access and support for QA tools and practices for sites that participate in NCI‐sponsored trials could vary greatly with sites that do not participate in these types of trials.

The results of our survey can be compared with best practice recommendations from the AAPM TG‐218 report, although it is important to remember that the TG‐218 report does not present the only acceptable solution. From our survey, approximately two of three sites use a composite measurement while the remainder utilize a field‐by‐field measurement. From TG‐218, the recommended delivery method for IMRT QA is a “true composite” followed by “perpendicular field‐by‐field”.^18^ The use of the perpendicular field‐by‐field is only recommended when true composite cannot be utilized because of the error‐prone nature of field‐by‐field evaluation.^18^ Our survey results indicate that the majority of institutions are consistent with this recommendation. Nevertheless, 17.9% of sites still utilize the “perpendicular composite” delivery method which utilizes a summed beam perpendicular to the measurement device for IMRT QA, which does not follow the TG recommendations. Utilizing point dose measurement for assessing agreement, TG‐218 recommends a tolerance of 2–3% while our survey shows that nearly half of the sites use looser criteria to assess agreement. In addition, TG‐51 Addendum states that ion chambers with volumes <0.05 cm^3^ are not recommended for reference dosimetry. While IMRT QA is not reference dosimetry, the goal is to measure absolute dose with high precision, and 23.8% of surveyed sites use microchambers for their IMRT QA.^19^

For planar and 3D/volumetric techniques that assess agreement, TG‐218 recommends to use absolute dose, not relative dose.^18^ From our survey, this seems to be followed by the large majority of sites, although approximately 10% of institutions still use relative dose to assess agreement for both planar and 3D/volumetric techniques. These results were self‐reported, and there is some ambiguity in regards to absolute vs relative techniques that our survey did not investigate. In particular, most array devices are calibrated relative to a user‐defined reference dose; doses are therefore typically relative to this input, and may not be absolute in the sense of direct traceability to NIST. Notable differences were also seen between clinical practice and the acceptance criteria recommended by TG‐218, which is to use ≥90% (action limits) or ≥95% (tolerance limits) of pixels passing with a 3%/2 mm gamma criteria.^18^ 96.4% of planar and 3D/volumetric techniques fell within the 90% action limits of pixels passing, while 60.7% and 58.1% fell within the tolerance limits of pixels passing respectively. While the passing rate range falls in line with the majority of sites, the gamma criteria in clinical practice was found to vary widely from site to site, with the majority of sites falling above the 2 mm threshold for both planar and 3D/volumetric techniques. Tighter criteria is recommended to further test the machines and regional errors, however, these tighter criteria were not utilized by respondents in the survey. It is of note that substantial inconsistencies have been reported in gamma pass rates through the various methods of sampling and interpolation used by different software calculations, as shown by Hussein et al.^20^ Further variations arise from the use of different user specifications in the calculation, such as local vs global differences and differences in noise or selection of reference vs evaluated datasets.^21^, ^22^, ^23^, ^24^, ^25^ This ambiguity may have a profound effect on the nature of IMRT QA and the apparent acceptability of plans for treatment.

For QA that does not pass criteria, several investigative strategies were provided and sites were allowed to rank their most common approach (1 denoting first strategy). Though most of our strategies are analogous (remeasure with the same setup, move to a new calculation point and remeasure, and replan) to the recommendations of TG‐218, several denote methods that fall outside of the standard practice. For example, the strategy of analyzing relative dose instead of absolute dose was used by over 25% of sites, with an average ranking of 2.8. As previously mentioned, TG‐218 recommends not to use relative dose. Another example was changing (loosening) the passing criteria for the case. This method was used by over 30% of sites with an average rank of 3.1. A majority of those sites already used looser criteria than what is recommended in TG‐218, and further loosening the criteria could cause important errors to be overlooked. Another strategy implemented by 17.4% percent of the institutions was to document and deliver the plan (average rank = 4.3). While an acceptable plan could fail IMRT QA, studies have shown that current IMRT QA methods have very high specificity,^8^, ^9^ meaning that if the plan is truly acceptable, IMRT QA devices will claim it is good. Plans that fail IMRT QA should therefore be managed with care. More generally, our survey shows that the strategies to handle failure of criteria is nonuniform, suggesting that the community as a whole is struggling with overcoming QA failures. Improving sensitivity and specificity of QA processes and devices could help alleviate some of the strain caused by these QA failures and create a more uniform approach to IMRT QA.

## 5 CONCLUSIONS

A survey was conducted across 1,455 radiotherapy sites, mostly located in the US and Canada, that examined several facets of 3D‐CRT and IMRT QA. For assessing agreement in IMRT QA, tools like the 2D diode array and point(s) measurement were the most common selection. The survey also found that array detector measurements vs calculation in phantoms dominated the type of comparison performed. These survey results represent a snapshot of IMRT QA practice, which is particularly timely given the recent recommendations from the AAPM TG‐218 report. The survey found that recommendations for patient‐specific IMRT QA were not universally implemented. 17.9% of sites still utilize a perpendicular composite delivery method to test IMRT QA, which is known to hide errors. In addition, a large proportion of sites still utilized looser criteria for all aspects of assessing agreement than what is recommended by TG‐218.^18^ Furthermore, our survey showed that strategies normally used when IMRT QA does not pass could potentially fall outside of current recommendations and that new strategies should be examined and implemented for better IMRT QA results.

## CONFLICTS OF INTEREST

The authors have no conflicts of interests to declare.

## Supporting information


**Data S1**. Complete survey.
