# Supplementary material for: Survey results of 3D‐CRT and IMRT quality assurance practice
Source: J Appl Clin Med Phys. 2020 Apr 30;21(7):70–6. doi: 10.1002/acm2.12885 (PMC7386182; doi:10.1002/acm2.12885)
Supplement: Supplementary file 1 — Data S1. Complete survey. [file ACM2-21-70-s001.pdf]

Appendix A: Complete Survey

Section 1:

Questions	Available Answers
Describe the method(s) used to conduct a check of the dose and monitor unit calculations generated by the 3DRTP system:	Open Ended
Are your 3D-CRT treatments monitored by a record and verify system?	Yes (Manufacturer & Model)<br>No

Section 2:

Questions	Primary Available Answers	Secondary Questions	Secondary Available Answers
Which of the following treatment modalities does you institution use? (Check all that apply)	Routine IMRT (Sliding Window, Step and Shoot, Tomotherapy etc.)	NA	NA
	VMAT/Rapid Arc	Is your QA for VMAT/Rapid Arc different from your routine IMRT QA?	Yes
			No
What are your standard tool(s) for verifying that the treatment unit delivers the planned dose for individual patients? (Choose all that apply.)	Point(s) Measurement	NA	Ion Chamber
			Diode
			TLD/OSLD
			Other
	Film	NA	NA
	2D Diode array	What comparison do you perform?	Measurement vs calculation in phantom
			Measurement mapped onto the patient CT dataset (e.g. DVH analysis)
	2D Ion Chamber array	What comparison do you perform?	Measurement vs calculation in phantom
			Measurement mapped onto the patient CT dataset (e.g. DVH analysis)
	EPID		
			Measurement vs calculation in phantom

	2.D (pseudo 3D) array/multi-plane array	What comparison do you perform?	Measurement mapped onto the patient CT dataset (e.g. DVH analysis)
	3D dosimeter	NA	NA
	Other	NA	NA

**Questions**	**Primary Available Answers**	**Secondary Questions**	**Secondary Available Answers**
When you make QA measurements, which of the following do you most commonly do?	Deliver beams at the same fixed gantry angle	NA	NA
	Deliver at the planned gantry angle		
Do you mount your detector on the gantry?	Yes	NA	NA
	No		
Are your plans usually assessed for pass or fail based on:	Each field-by-field measurement	NA	NA
	Composite measurement (all fields)		
How do you assess agreement (select all that apply), and what are your most commonly used comparison criteria?	Point Dose	Typical number of points analyzed?	1
			2-3
			>3
		What is your acceptance passing criterion (%)?	Open Ended
	Planar	When you analyze your measurement do you use:	Absolute dose
			Relative dose
		Typical number of planes analyzed (not fields):	1 plane
			2 – 3 planes
			>3 planes
		What test do you perform?	Gamma Analysis (___%/____mm agreement and ___% pixels passing criteria)
			Qualitative evaluation

			Other
	3D/Volumetric analysis	When you analyze your measurement do you use:	Absolute dose
			Relative dose
		Typical number of planes analyzed (not fields):	1 plane
			2 – 3 planes
			>3 planes
		What test do you perform?	Gamma Analysis (___%/____mm agreement and ___% pixels passing criteria)
			Qualitative evaluation
			Other

**Questions**	**Primary Available Answers**
Do you do routine in-vivo dosimetry for IMRT patients?	Yes<br>No
If your QA does not meet your passing criteria, what actions do you take? (choose all that apply, rank in order of attempt (1 denotes first strategy))	Re-measure with the same setup (at the same point/plane)
	Move to a new calculation point/plane and re-measure
	Try fixed gantry angle delivery
	Re-plan
	Scale the MU's (partially or fully)

	Change the passing criteria for the case
	Analyze in relative dose mode instead of absolute dose mode
	Document result and deliver the plan
	Something else: ___________
Additional Comments	Open Ended
